# Predictors of the Sense of Embodiment of a Female Victim of Sexual Harassment in a Male Sample Through 360-Degree Video-Based Virtual Reality

**DOI:** 10.3389/fnhum.2022.845508

**Published:** 2022-05-06

**Authors:** Sara Ventura, Marta Miragall, Georgina Cardenas, Rosa M. Baños

**Affiliations:** ^1^Department of Psychology, University of Bologna, Bologna, Italy; ^2^Instituto Polibienestar, University of Valencia, Valencia, Spain; ^3^Department of Personality, Evaluation, and Psychological Treatments, University of Valencia, Valencia, Spain; ^4^The Spanish Biomedical Research Centre in Physiopathology of Obesity and Nutrition (CIBERObn), Instituto de Salud Carlos III, Madrid, Spain; ^5^Laboratorio de Enseñanza Virtual y Ciberpsicologıa, Facultad de Psicología, Universidad Nacional Autónoma de México, Ciudad de México, Mexico

**Keywords:** body-swap illusion, 360-degree video, virtual reality, embodiment, sexual harassment

## Abstract

The sense of embodiment refers to the set of sensations related to having (i.e., ownership), being located in (i.e., location), and controlling (i.e., agency) a virtual body. Recently, 360-degree video-based Virtual Reality (VR) has been used to manipulate the sense of embodiment, generating the body-swap illusion, that is, the illusionary switch from the real body to a virtual one. However, the psychological mechanisms involved in this illusion are still unknown. The present study is a secondary analysis of the study by Ventura et al. (2021) investigating the feasibility of 360-degree video to induce the body swap from a male’s real body to a female virtual body in a sexual harassment virtual environment. In addition, the study explores whether the sense of presence and psychological trait variables related to sexual harassment (i.e., machismo, chivalry, alexithymia, empathic abilities) predict the illusion of owning the body of a female victim of sexual harassment. Forty-four men participated in the study, and the results indicate that the 360-degree video is able to induce the body-swap illusion for location and ownership, but not for agency. Multiple regression analyses showed that the sense of presence was a predictor of the three dimensions of embodiment, but specific psychological traits (i.e., low scores on machismo, high scores on difficulties expressing feelings, and high scores on perspective taking) were also predictor variables of experiencing a greater sense of location and agency in the female virtual body. This study shows that both technological issues and participants’ psychological traits are involved in the experience of the body-swap illusion in a sexual harassment scenario using 360-degree video-based VR.

## Introduction

The body is represented in the brain by integrating internal (e.g., memory) and external (e.g., the surrounding environment) input. This representation is the sense of embodiment, which consists of three subcomponents: (i) the sense of body ownership (the sense of one’s self-attribution of a body, or self-identification); (ii) the sense of agency (the sense of causing the body’s action); and (iii) the sense of self-location (the feeling of being an entity located in a position in space and perceiving the world from that position and perspective) (Blanke and Metzinger, 2009; Kilteni et al., 2012).

In the past decade, several studies have demonstrated the ability of Virtual Reality (VR) to induce body-swap illusions by altering the sense of embodiment. The body-swap illusion is defined as the illusion of having another body, in terms of ownership, agency, and location (Ehrsson et al., 2005; Slater et al., 2010; Kilteni et al., 2012). The body-swap illusion can be induced through advanced VR systems, such as optical sensors that track body movements that are later displayed through a Head Mounted Display (Oh et al., 2016), or the optitrack suit, which displays the body movements in the virtual scenario and allows users to see themselves with another body (Peck et al., 2013). Another system that allows the body-swap illusion is 360-degree video-based VR (Ventura et al., 2022). It is a low-cost immersive technology -compared to other technologies on the market- that records in every direction simultaneously (Huang et al., 2017; Repetto et al., 2018). Then, the resulting 360-degree video can be displayed through a Head Mounted Display. The users view the recorded environment all around them, as in real life, but without interactivity, due to the video’s predetermined technological features (Jun et al., 2020). Furthermore, the 360-degree video has been shown to induce a high sense of presence (Aitamurto et al., 2018), that is, the feeling of being “there” in the virtual environment (Usoh et al., 2000).

One of the main characteristics of VR systems is their ability to induce the body-swap illusion from the real participant’s body to an artificial avatar’s body (Kilteni et al., 2012). Previous studies have adopted the body-swap illusion to switch the participant’s gender from male to female (Slater et al., 2010; Gonzalez-Liencres et al., 2020), the skin color from white to black (Peck et al., 2013; Banakou et al., 2020), or the age from younger to older (Banakou et al., 2013; Oh et al., 2016). The body-swap illusion, if successfully achieved by participants, can elicit changes in interpersonal attitudes (Peck et al., 2013). For example, previous studies have shown that achieving virtual alterations in age by embodying an older person can reduce negative stereotypes toward the elderly (Oh et al., 2016), and virtual alterations in skin tone from a light-skinned participant to a dark-skinned avatar can produce a significant reduction in implicit biases toward dark-skinned people (Peck et al., 2013; Banakou et al., 2020). In this line, Seinfeld et al. (2018) found that the body-swap illusion -from a male real body to a female virtual body victim of intimate partner violence- significantly improved the male participants’ performance on an emotion recognition task (i.e., increased ability to recognize female fearful faces and reduced bias in recognizing fearful faces as happy). Similarly, Neyret et al. (2020) found that male participants who embodied a female avatar victim of harassment reduced their behavioral conformity during Milgram’s Obedience experiment 1 week later (i.e., men who had embodied a female avatar 1 week earlier gave fewer shocks to a woman on the obedience task compared to controls). Moreover, Gonzalez-Liencres et al. (2020) conducted a study of intimate partner violence in VR, and they found that the level of identification with the female avatar correlated with the decrease in prejudice against women after exposure to VR. Another study demonstrated that the 360-degree video could be an effective tool to increase male empathy and decrease violent attitudes while embodying a female victim of sexual harassment (Steinfeld, 2020).

To date, the role of the psychological traits involved in enhancing a body-swap illusion during experiments has been poorly examined (Dewez et al., 2019). However, previous findings from studies on creating body-swap illusions showed that some participants easily experienced the illusion, whereas other participants were more resistant to perceiving themselves with another body (Dewez et al., 2019). On this topic, Shin (2018) carried out a study to explore the variables involved in the participants’ experience and psychological outcomes in a 360-degree video experiment. The author found that the sense of presence was an important predictor of the sense of embodiment and empathy, which in turn led to greater engagement with the VR experience. In addition, other psychological traits (e.g., higher absorption and more willing-to-be-immersed personalities) of users could also influence the sense of embodiment (Shin, 2018). However, the study of the interaction between the sense of presence, the participant’s psychological traits, and the sense of embodiment in the field of 360-degree video is scarce.

The present study is a secondary analysis of the study by Ventura et al. (2021), which had a within-subjects design in which 44 men experienced the daily life of a female victim of sexual harassment in two conditions: a 360-degree video-based VR experience vs. a traditional perspective-taking task.^1^ The 360-degree video-based VR camera experience began with an explicit induction of embodiment in a female body (i.e., the female virtual body doing some movements with her arms and legs that men had to follow). Then, several sexual harassment scenarios that took place in Mexico City were displayed (e.g., harassing behavior at the university, at home with the spouse of the victim, in the subway). Self-reported questionnaires that measured the traits of machismo and chivalry, empathic abilities, and alexithymia were administered before the VR experience, whereas the sense of presence, cyber-sickness, and embodiment were measured after the VR experience. Results showed the superiority of the VR experience in increasing empathy, sense of oneness, and perspective-taking toward a female victim of sexual harassment.

In this study, we investigated whether the embodiment of the female body might be influenced by participants’ personality traits (machismo, chivalry empathy, alexithymia) and their level of sense of presence in the virtual environment in a sample of 44 male participants. This question arises from previous evidence linking personality traits (e.g., openness to experience) to immersive tendencies (e.g., being more or less responsive to the virtual environment) (Weibel et al., 2010), and linking this immersive tendency to the sense of presence (Servotte et al., 2020). Furthermore, the evidence also shows that the sense of embodiment may be lower in hostile scenarios (Steed et al., 2018). Men with high machismo, chivalry, and difficulties with alexithymia and empathy may perceive a sexual harassment environment as more hostile because these variables have been strongly related to violent sexual behavior (Locke and Mahalik, 2005; Teten et al., 2008). Therefore, participants with these characteristics may have a lower immersive tendency in this context, which may interfere with their sense of presence and their sense of embodying a female victim of sexual harassment. In this study, we aimed to disentangle whether these psychological traits are involved in enhancing or interfering with the experience of sensing one’s body as a female virtual body.

The main objectives were: (1) to confirm the feasibility of the 360-degree video for generating both the sense of presence and the sense of embodiment (i.e., the body-swap illusion from a male body to a female body); and (2) to analyze whether the sense of presence and some psychological traits associated with sexual harassment (i.e., machismo, chivalry, alexithymia, empathic abilities) are predictors of the sense of embodiment.

We hypothesized that: (1) the 360-degree video would generate a significant body-swap illusion from the male’s real body to a female virtual body in terms of location and ownership, but not in terms of agency (given the predetermined movements of the avatar with this technology; Jun et al., 2020); and (2) the sense of presence would predict the sense of embodiment (based on the literature supporting this relationship, e.g., Shin, 2018), but the participants’ psychological trait variables would also be involved in the effect of the sense of embodiment. We did not specify which variables would be predictors of the sense of embodiment, given the exploratory nature of this study. The present study would confirm whether some relevant psychological trait variables involved in sexual harassment—in addition to the sense of presence—might cause men’s resistance or predisposition to being embodied in a female victim’s virtual body. These findings will increase the effectiveness of psychological interventions designed to change interpersonal attitudes based on the body-swap illusion.

## Materials and Methods

The procedures and materials were approved by the Ethics Committee of the Universidad Nacional Autónoma de México (UNAM) with the code EP/PMDPSIC/0151/19.

### Participants

Participants were recruited through advertisements at UNAM (Mexico City) and in social networks. The inclusion criterion was being a man older than 18 years old. The exclusion criteria were: (1) having physical problems that could inhibit free movements (e.g., back or neck pain); (2) a history of sexual harassment with legal consequences; (3) use or abuse of drugs; and (4) receiving psychological treatment at the time of the study.

A convenience sample of 44 male participants took part in the study. They were all volunteers, and they signed the informed consent document before starting the experiment, in accordance with the Declaration of Helsinki.

### Measures

#### Sociodemographic Questionnaire

This is an *ad hoc* questionnaire that contains 10 items that collect information about age, sex, education level, history of mental or chronic illness, use or abuse of drugs, alcohol consumption, current psychological treatments, and whether the participants have/had legal problems due to sexual harassment.

#### Empathic Abilities

The Interpersonal Reactivity Index (Davis, 1980; Pérez-Albéniz et al., 2003) is a self-report containing 28 items that measure: (1) perspective taking (ability to adopt the viewpoint of another person); (2) fantasy (ability to transpose oneself into the feelings of a fictitious character); (3) empathic concern (feeling of being involved in others’ emotions); and (4) personal distress (feelings of sorrow about others’ pain). Items are rated on a 7-point Likert scale (1 = strongly disagree; 7 = strongly agree), where a higher score indicates greater empathic abilities. The mean for each subscale was calculated (scores ranged from 1 to 7). In this study, the internal consistency values were: α = 0.60 (for perspective taking), α = 0.74 (for fantasy), α = 0.67 (for empathic concern), and α = 0.83 (for personal distress).

#### Alexithymia

The Toronto Alexithymia Scale (TAS-20) (Taylor et al., 1985; Moral de la Rubia, 2008) is a self-report scale containing 20 items that measure: (1) difficulty expressing feelings, (2) difficulty identifying feelings, and (3) externally oriented thinking. Items are rated on a 7-point Likert scale (1 = strongly disagree; 7 = strongly agree), where a higher score indicates higher alexithymia. The mean for each subscale was calculated (scores range from 1 to 7). In this study, the internal consistency values were: α = 0.84 (for difficulty expressing feelings), α = 0.91 (for difficulty identifying feelings), and α = 0.51 (for externally oriented thinking).

#### Machismo and Chivalry

The Machismo and Chivalry Scale (Arciniega et al., 2008) is a self-report scale containing 20 items that measure the constructs of “machismo” (referring to power and men’s aggressive attitudes) and chivalry (referring to emotional connectedness, honor, and men’s nurturance). The items are rated on a 7-point Likert scale (1 = strongly disagree; 7 = strongly agree), where a higher score indicates higher levels of machismo and chivalry. The mean for each subscale was calculated (scores ranged from 1 to 7). In this study, internal consistency was adequate for the two subscales: machismo (α = 0.80) and chivalry (α = 0.78).

#### Embodiment, Presence, and Sickness

##### Sense of Embodiment, Sense of Presence, and CS Scale

This is a self-report questionnaire with 16 items rated on a 7-point scale (1 = strongly disagree; 7 = strongly agree). Ten items were adapted from Longo’s original questionnaire to assess the ownership, location, and agency of the female body (Longo et al., 2008) (α = 0.88); three items were developed by the authors to assess the sense of presence felt during the whole immersive experience (Usoh et al., 2000) (α = 0.46); and three items were developed by the authors to detect the CS level of the participant during the VR experience (Kennedy et al., 1993) (α = 0.87). The mean for each construct was calculated (scores ranged from 1 to 7).

### Implementation and Apparatus

The 360-degree video was recorded with an LG360-105 camera and the LG360 viewer software, and it was edited with the Adobe Premiere Program. The video contains a short story about a female victim of sexual harassment from her point of view, designed to induce the participant to take the victim’s perspective. The scenarios were recorded in Mexico City, where actors performed several harassment situations that reflect a typical day of a victim of sexual harassment (Ventura et al., 2021). To allow the first-person perspective, the female actor held the 360-degree video on her head with a head strap during the entire video. The video lasted almost 25 min, and it was uploaded to YouTube and played on an iPhone 6 with the VR option, supported by a VR GLASS FOV: 120° to create the VR immersion experience.

**FIGURE 1:**
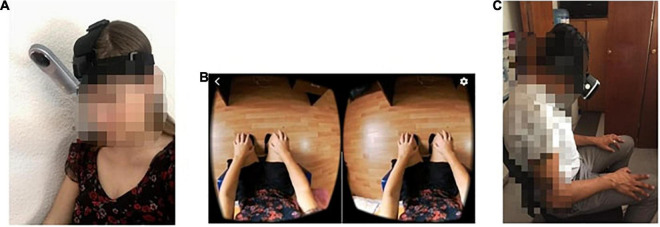
The 360-degree video to induce the body-swap illusion. **(A)** Recording the video. **(B)** Female participant’s perspective. **(C)** Male participant doing the experiment.

Before the sexual harassment video was presented, the body swap illusion was induced. As in Ventura et al., 2022, who showed that imitating slow movements allows participants to better synchronize their limbs with the performer’s, the body swap illusion was induced by imitating movements. To this end, an embodiment induction task was created by recording a female actress doing slow movements with her limbs while sitting on a chair (e.g., moving her hands up and down, rubbing her limbs, and rotating her hands, see Figure 1).

Once they had become immersed in the headset, the participants completed the embodiment induction exercise. Then, they experienced the SH 360° video.

### Experimental Procedure

The experiment took place at the Laboratorio de Enseñaza Virtual y Ciberpsicologia (Mexico City). After giving their consent, participants filled out the sociodemographic, interpersonal reactivity index (IRI) (empathy), TAS-20 (alexithymia), and MSC (machismo and chivalry) questionnaires. Then they were instructed to sit comfortably on a chair with their legs resting on a footstool and their arms resting on their legs. Before starting the video, the researcher gave them the instruction to imitate the body movements of the female performer they would see on the video as closely as possible during the entire experiment. Moreover, participants could quit the experiment if they felt sick, and people wearing glasses could keep them on if they did not feel discomfort. During the experimental session, participants wore the Head Mounted Display, and they were left alone in the room while doing the embodiment exercises. When the video ended, the participants were invited to fill out the embodiment questionnaire and ask any questions they might have about the study.

## Data Analyses

All the statistical analyses were performed using SPSS v.26. First, descriptive statistics were calculated for the sociodemographic data and the psychological traits. Second, the assumption of normality was checked with Shapiro-Wilk to decide which test should be computed. One-sample *t*-tests (parametric test) or one-sample Wilcoxon Signed-rank tests (non-parametric test) were conducted to explore whether the effects of the 360-degree video on the sense of embodiment scores and the sense of presence were significantly different from the level of chance of 4 (on a scale from 1 to 7). Third, three multiple regression analyses were performed to test whether the psychological trait measures (*MCS, IRI, TAS-20*) predicted the embodiment scores after controlling for the sense of presence. The sense of presence was entered in the first step of the multiple regression, whereas the psychological trait measures were entered in the second step. All the predictor variables were entered in the second block using the stepwise method, given that we did not have an *a priori* hypothesis about which specific independent variables would predict the dependent variables. Finally, we tested the absence of multicollinearity using the Variance Inflation Factor (VIF). Two-tailed significance tests were considered at *p* < 0.05. Finally, using G*power v.3.1.9.7, we performed a *post hoc* power analysis to determine whether the secondary analyses in this study had enough power to detect effects greater than or equal to *d* = 0.40 i.e., the standard effect size in Psychology, according to Brysbaert (2019). Results showed that this study had 82.54, 72.66, and 65.18% power to detect an increase in the explained variable in a multiple linear regression testing 1, 2, and 3 additional predictor variables over one predictor variable (i.e., sense of presence), respectively.

## Results

### Descriptive Statistics for Sociodemographic and Psychological Trait Measures

Descriptive statistics for sociodemographic (i.e., age, education, history of mental, and chronic illness, and history of legal consequences for sexual abuse) and psychological trait measures of machismo and chivalry (MCS), empathic abilities (IRI), and alexithymia are shown in Table 1.

**TABLE 1:** Descriptive statistics of sociodemographic and psychological trait measures (*N* = 44).

	*M (SD)*	%
**Age**	26.20 (8.36)	–
**Educational level**		
Secondary studies	–	6.8%
Degree	–	77.3%
Master	–	15.9%
**History of mental or chronic illness (% yes)**	–	9.1%
**Alcohol consumption**		
Never	–	25.0%
Once per month	–	36.4%
2–4 times per month	–	34.1%
>2–3 times a week	–	4.5%
**Machismo and chivalry (MCS)**		
Machismo	2.01 (0.84)	–
Chivalry	5.36 (0.97)	–
**Empathetic abilities (IRI)**		
Perspective taking	4.78 (0.77)	–
Fantasy	4.66 (0.99)	–
Empathy concern	5.17 (0.81)	–
Personal distress	3.15 (1.13)	–
**Alexithymia (TAS-20)**		
Difficulty in express feelings	3.67 (1.46)	–
Externally oriented thinking	2.49 (0.73)	–
Difficulty in identify feelings	2.93 (1.47)	–

*MCS, Machismo and Chivalry Scale; IRI, Interpersonal Reactivity Index; TAS-20, Toronto Alexithymia Scale.*

### Sense of Embodiment and Sense of Presence During the 360-Degree Video

Means and standard deviations for each item are presented in Table 2. A one-sample *t*-test indicated that the sense of embodiment scores were not significantly greater than the chance level of 4 for agency (*M* = 4.28, *SD* = 1.50), *t*(43) = 1.24, *p* = 0.222. Two one-sample Wilcoxon Signed-rank tests indicated that the sense of embodiment scores were not significantly greater than the chance level of 4 for location (*M* = 5.82, *SD* = 1.33), *Ws* = 833.00, *z* = 4.86, *p* < 0.001, and ownership (*M* = 5.12, *SD* = 1.10), *Ws* = 882.00, *z* = 4.97, *p* < 0.001.

**TABLE 2:** Descriptive statistics for each item on the embodiment and sense of presence scales (*N* = 44).

Item	Factor	*M (SD)*
1. I felt as if I was looking at myself.	Ownership	4.98 (1.47)
2. I experienced the arms of the performer as my own arms.	Ownership	5.45 (1.27)
3. I experienced the legs of the performer as my own legs.	Ownership	5.20 (1.37)
4. I experienced the body of the performer as my own body.	Ownership	5.32 (1.27)
5. I had the feeling to have another body.	Ownership	4.64 (1.54)
6. I had the illusion of sitting in the same place of the performer.	Location	5.82 (1.33)
7. I felt I had control over the arms of the performer.	Agency	4.36 (1.57)
8. I felt I had control over the legs of the performer.	Agency	4.20 (1.58)
9. I felt I had control over the body of the performer.	Agency	4.27 (1.63)
10. I had the feeling to have a female body.	Ownership	5.14 (1.61)
11. I had the illusion to be there, in the virtual environment.	Presence	6.00 (0.94)
12. I was confused if the environment was real, or a video recorded	Presence	4.07 (1.92)
13. When I think to the scenario, I have the feeling that I was there	Presence	5.64 (1.26)

**FIGURE 2:**
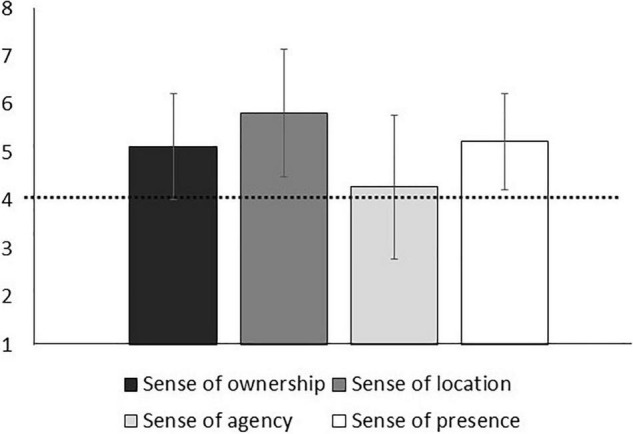
Graphical representation of the sense of embodiment and sense of presence scales (*N* = 44). Error bars represent 1 standard deviation from the mean, and the dashed line represents the mean value of the scale (ranging from 1 to 7).

Scores for sense of presence (*M* = 5.23, *SD* = 1.00) were also greater than the chance level of 4, *t*(43) = 8.23, *p* < 0.001 (Figure 2).

### Multiple Regression Analyses: Is Sense of Embodiment Predicted by Sense of Presence, Machismo and Chivalry, Empathic Abilities, and Alexithymia?

**FIGURE 3:**
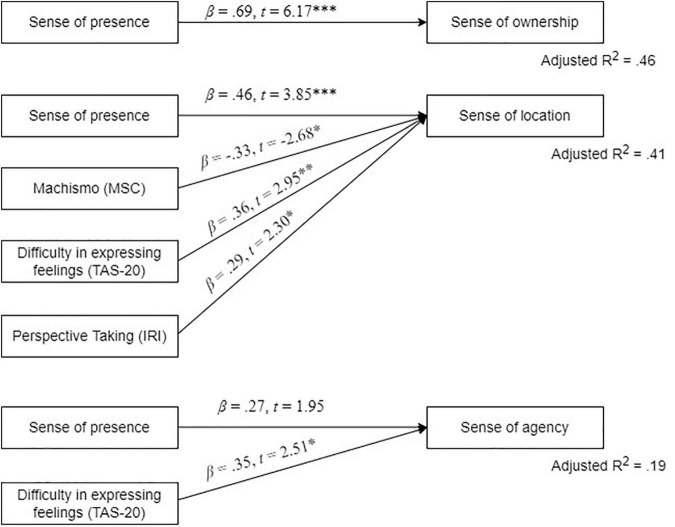
Graphical representation of the stepwise multiple regressions of embodiment scores. MCS, Machismo and Chivalry Scale; IRI, Interpersonal Reactivity Index; TAS-20, Toronto Alexithymia Scale.

Coefficients of determination, unstandardized coefficients, standard errors, standard coefficients, and *t*-statistics for the three stepwise multiple regression analyses are shown in Table 3 and Figure 3. The Variance Inflation Factor ranged from 1.021 to 1.170 for all the regression analyses, indicating that there were no problems with multicollinearity.

**TABLE 3:** Stepwise multiple regressions of embodiment scores.

Outcomes	Predictors	*R*	Adjusted *R*^2^	*R*^2^ Change	*B*	*SE*	β	*t*
**Ownership**	**Model 1**							
	Constant				1.15	0.66		1.75
	Sense of presence	0.69	0.46	0.48	0.76	0.12	0.69	6.17***
**Location**	**Model 1**							
	Constant				2.80	1.00		2.81**
	Sense of presence	0.43	0.17	0.19	0.58	0.19	0.43	3.09**
	**Model 2**							
	Constant				3.69	1.00		3.71***
	Sense of presence				0.61	0.18	0.46	3.48***
	Machismo (MSC)	0.55	0.27	0.11	-0.54	0.21	-0.34	-2.59*
	**Model 3**							
	Constant				3.13	0.96		3.24**
	Sense of presence				0.56	0.17	0.42	3.33**
	Machismo (MSC)				-0.64	0.20	-0.41	-3.22**
	Difficulty in expressing feelings (TAS-20)	0.63	0.35	0.10	0.29	0.12	0.32	2.50*
	**Model 4**							
	Constant				-0.02	1.65		-0.01
	Sense of presence				0.62	0.16	0.46	3.85***
	Machismo (MSC)				-0.53	0.20	-0.33	-2.68*
	Difficulty in expressing feelings (TAS-20)				0.33	0.11	0.36	2.95**
	Perspective Taking (IRI)	0.68	0.41	0.07	0.51	0.22	0.29	2.30*
**Agency**	**Model 1**							
	Constant				1.74	1.17		1.48
	Sense of presence	0.32	0.08	0.10	0.49	0.22	0.32	2.20*
	**Model 2**							
	Constant				0.82	1.17		0.71
	Sense of presence				0.41	0.21	0.27	1.95
	Difficulty in expressing feelings (TAS-20)	0.47	0.19	0.12	0.36	0.14	0.35	2.51*

*p < 0.05; **p < 0.01; ***p < 0.001. MCS, Machismo and Chivalry Scale; IRI, Interpersonal Reactivity Index; TAS-20, Toronto Alexithymia Scale. R, Multiple Correlation Coefficient; R^2^, Coefficient of determination; R^2^ Change, Coefficient of determination Change; B, Unstandardized coefficient; SE, Standard Error; β, Beta coefficient; t, t statistic (estimated coefficient divided by its own SE).

The first multiple regression analysis, to predict ownership, showed that *sense of presence* was a significant positive predictor. This model was statistically significant, *F*(1, 43) = 38.04, *p* < 0.001, *R*^2^ = 0.463, explaining 46.3% of the variance (Model 1). None of the psychological trait variables entered in the second step were significant (*p* > 0.05).

The second multiple regression analysis, to predict location, showed that *sense of presence* was a significant positive predictor. The model was statistically significant, *F*(1, 43) = 9.53, *p* = 0.004, *R*^2^ = 0.165, explaining 16.5% of the variance (Model 1). After introducing the psychological trait variables in the second step of the multiple regression, three other models were also significant: *F*(2, 43) = 8.76, *p* < 0.001, *R*^2^ = 0.265 (Model 2), *F*(3, 43) = 8.67, *p* < 0.001, *R*^2^ = 0.349 (Model 3), and *F*(4, 43) = 8.52, *p* < 0.001, *R*^2^ = 0.412 (Model 4). In Model 2, machismo (MSC) was a significant negative predictor of location that explained an additional 11.4% of the variance, whereas *sense of presence* remained significant. In Model 3, *difficulty expressing feelings* (TAS-20) was a significant positive predictor of location that explained an additional 9.5% of the variance, whereas the *sense of presence* and *machismo* remained significant. Finally, in Model 4, *perspective-taking capacity* (IRI) was a significant positive predictor of location that explained an additional 7.2% of the variance, whereas *sense of presence*, machismo, and difficulty expressing feelings remained significant. The linear combination of the *sense of presence* and these psychological trait variables was the best fitting model, explaining 41.2% of the variance. None of the other psychological trait variables (i.e., chivalry, difficulty identifying feelings, externally oriented thinking, empathic concern, personal distress, and fantasy) entered in the second step were significant (*p* > 0.05).

The third multiple regression analysis, to predict agency, showed that the *sense of presence* was a significant predictor of the model. The model was significant, *F*(1, 43) = 4.85, *p* = 0.033, explaining 8.2% of the variance (Model 1). After introducing the trait variables in the second step of the multiple regression, a second model was significant (Model 2). In Model 2, *difficulty expressing feelings* (TAS-20) was a significant positive predictor that explained an additional 12.0% of the variance. The model was significant, *F*(1, 43) = 5.89, *p* = 0.006, explaining 18.5% of the variance. However, the *sense of presence* was marginally significant in Model 2, β = 0.27, *t* = 1.95, *p* = 0.059. None of the other psychological trait variables (i.e., machismo and chivalry, difficulty identifying feelings, externally oriented thinking, perspective-taking capacity, empathic concern, personal distress, and fantasy) entered in the second step were significant (*p* > 0.05).

## Discussion

The main objectives of the present study -which is a secondary analysis of Ventura et al. (2021)- were: (1) to confirm the ability of the 360-degree video-based VR camera to alter the sense of embodiment by inducing a body-swap illusion from a male’s real body to a female virtual body in terms of location, ownership, agency, and sense of presence; and (2) to analyze whether psychological trait variables associated with sexual violence – in addition to the sense of presence – predicted the sense of embodiment. These objectives were tested in a sample of Mexican men who were placed in a sexual harassment scenario using 360-degree video-based VR technology. The 360-degree video began with a body swap task (i.e., the female virtual body doing some movements with her arms and legs that the men had to follow). Then, several sexual harassment scenarios in Mexico City were displayed (e.g., harassing behavior at the university, at home with the spouse of the victim, in the subway). Self-reports to measure the psychological traits of machismo and chivalry, empathic abilities, and alexithymia were administered before the VR experience, whereas the sense of presence and sense of embodiment were measured after the VR experience.

Results showed that participants experienced a significantly high sense of location and ownership of the female body experiencing sexual harassment, but not a high sense of agency. The sense of presence during the exposure was also significantly high, which is in line with previous work that highlighted the ability of the 360-degree video to induce a high sense of presence (Breves and Heber, 2020). Hence, the first hypothesis was supported, given that the VR experience induced the illusion of owning a female body (i.e., ownership) and perceiving their own body in the virtual scenario (i.e., location), that is, the feeling of being there, but it did not promote the illusion of moving the female virtual body (i.e., agency). These findings confirm previous literature that concluded that synchronized movements between the participant and the virtual avatar are crucial to inducing the body-swap illusion (Petkova and Ehrsson, 2008), especially in the case of embodying a virtual body that is different from the participant’s body in terms of sex, race, or age (Peck et al., 2013; Oh et al., 2016).

The second hypothesis was also supported. There were significant effects of the sense of presence and some psychological trait variables on the sense of embodiment. Regarding the sense of presence, it was a significant positive predictor of the three dimensions of embodiment, explaining 46.3% of variance in the sense of ownership, 18.5% in the sense of location, and 8.2% in the sense of agency. As in previous studies (Aitamurto et al., 2018; Dewez et al., 2019), we found support for the proposal that a high sense of presence positively predicts the sense of embodiment in terms of ownership, location, and agency; that is, the more immersive the system is, the more likely users are to feel present in the virtual environment and perceive themselves as having a virtual body (Shin, 2018; Borrego et al., 2019). However, in addition to the sense of presence, some relevant psychological traits of users were also predictive variables of the sense of embodiment. On one hand, lower scores on machismo, higher scores on difficulty expressing feelings, and higher scores on perspective-taking predicted higher scores on the sense of location (11.4, 9.5, and 7.2% of the variance, respectively). On the other hand, higher scores on difficulty expressing feelings predicted higher scores on the sense of agency, accounting for 12% of the explained variance. However, the factors of *empathic concern*, *personal distress*, and *fantasy* were not significant predictors in the models, perhaps because they require more cognitive and emotional effort and the immersive experience did not last long enough to elicit a deep empathic response.

Hence, men with lower scores on machismo and difficulties expressing feelings and a greater ability to adopt another person’s viewpoint had a higher illusion of being in the female virtual body. First, machismo stood out as an important predictor variable, given that a lower score on this variable predicted a higher illusion of feeling located in the female virtual body. Machismo can be defined as a strong sense of masculinity (Hurtado and Sinha, 2016) associated with a low ability to take the perspective of women (Mayer et al., 2018). Previous studies have found that the illusion of “being in a rendered space and that the unfolding events were really happening” can be impeded if the VR scenario is perceived as hostile (e.g., Steed et al., 2018). Thus, in the present study, some participants might have experienced the scenario as unpleasant because it was not consistent with their masculinity-related values and did not reflect their perception of women’s reality. Therefore, future studies should take into account that entering this virtual environment may be an unpleasant experience, and that this discomfort may act as an obstacle to feeling the body-swap illusion. Second, alexithymia was another significant predictor variable of sense of embodiment because a lower ability to express emotions was associated with a higher illusion of moving the female virtual body (i.e., sense of agency). This could be related to the fact that alexithymia has been associated with low interoceptive awareness (i.e., the ability to detect, accurately monitor, and regulate internal bodily processes) (Brewer et al., 2016; Murphy et al., 2018; Trevisan et al., 2019), which, in turn, has been related to greater susceptibility to the embodiment of other bodies, as occurs in eating disorders (Tsakiris et al., 2011; Eshkevari et al., 2012; Herbert and Pollatos, 2012). Future studies should test whether low interoceptive awareness is the mediator variable that could explain the increased sense of embodiment in men who have difficulties expressing their feelings. Third, it should be highlighted that participants’ perspective-taking ability elicits the illusion of being located in the virtual environment. Thus, a higher cognitive awareness of the woman’s state allowed them to experience a greater sense of embodiment.

Overall, low machismo, willingness to understand the woman’s perspective, and high alexithymia are the psychological traits most favorable to experiencing the sense of embodiment of a female body in a sexual harassment context. These findings are aligned with previous studies that pointed to the influence of the sense of presence and the user’s psychological traits on the quality of the VR experience (Shin, 2018; Shin and Biocca, 2018). Future studies should consider that the sense of embodiment depends not only on technical and procedural issues (e.g., high quality of the apparatus and the extent to which the movements are synchronized), but also on cognitive attitudes and beliefs and affective processes. In this regard, men who were more predisposed to understanding women and had less prejudice toward women experienced the body-swap illusion to a greater extent. Consequently, prevention and rehabilitation interventions for male offenders based on the body-swap illusion should be administered only after the required traits (i.e., machismo, alexithymia, perspective taking) have been cognitively restructured. This would make it easier for men to embody a woman’s body, thus increasing the success and benefits of the body-swap illusion. In this regard, a recent systematic review conducted by Travers et al. (2021) pointed to the need to identify the specific components that should be targeted to avoid recidivism in intimate partner violence. Therefore, our findings may identify relevant therapeutic targets to be considered in future interventions.

Limitations of this study should be noted. The first weakness is related to the fact that the results are only generalizable to a sample of young Mexican men who were motivated to participate in a study related to understanding the experience of being a woman. The second limitation has to do with the low internal consistency of the *externally oriented thinking* subscale of the TAS-20 (α = 0.51) and the sense of presence scale (α = 0.46), and so the results should be interpreted with caution. The third limitation has to do with the apparatus itself. As we expected, the low scores on the sense of agency could be explained by the fact that the 360-degree video was pre-recorded, which meant that participants simply followed the movements of the female performer, without having the freedom to make their own movements. Finally, there is no information about the mid- and long-term effects of experiencing high embodiment on violence and sexual harassment toward women in the participants’ daily lives. That is, future studies should investigate whether the changes in empathy and related variables found in the primary analyses of this study (Ventura et al., 2021) last over time. Hence, future studies should test whether this higher sense of embodiment leads to more significant changes in attitudes (e.g., lower attitudes about the acceptability of such violence) or improved psychological traits related to the perpetration of violence (e.g., higher empathy), in both the perpetration and prevention of sexual harassment. By confirming this, a “target” profile of men for this kind of VR-based intervention could be created, in order to guarantee success in the therapeutic goal of reducing or preventing sexual harassment.

The current study shows that 360-degree video-based VR is a potential tool to induce embodiment whose key components are the sense of presence and the user’s cognitive and affective traits. More specifically, the sense of presence was a predictor of the three dimensions of embodiment, but specific psychological traits (i.e., men with low machismo, willing to understand the woman’s perspective, but with difficulties expressing their feelings) were also predictor variables of experiencing a greater sense of location and agency in the female virtual body in a sexual harassment scenario. This study shows that both technological issues and participants’ psychological traits are involved in the experience of embodiment in a sexual harassment scenario using 360-degree video-based VR.

## Data Availability Statement

The raw data supporting the conclusions of this article will be made available by the authors, without undue reservation.

## Ethics Statement

The studies involving human participants were reviewed and approved by the Universidad Nacional Autonoma de Mexico (Mexico) with the code EP/PMDPSIC/0151/19. The patients/participants provided their written informed consent to participate in this study.

## Author Contributions

SV and RB made substantial contributions to the conceptualization, design of the experiment, formal analyses, collection of the data, and drafting the manuscript. GC made substantial contributions to data collection. MM made substantial contributions to the formal analyses and drafting the manuscript. RB made substantial contributions to revising the manuscript critically for important intellectual content. All authors provided final approval of the version to be published and agreed to be accountable for all aspects of the work and ensure that questions related to the accuracy or integrity of any part of the work are appropriately investigated and resolved.

## Conflict of Interest

The authors declare that the research was conducted in the absence of any commercial or financial relationships that could be construed as a potential conflict of interest.

## Publisher’s Note

All claims expressed in this article are solely those of the authors and do not necessarily represent those of their affiliated organizations, or those of the publisher, the editors and the reviewers. Any product that may be evaluated in this article, or claim that may be made by its manufacturer, is not guaranteed or endorsed by the publisher.

## Footnotes

1 Participants in this study went through two conditions (i.e., VR and narrative task without VR). However, because we followed a within-subjects design and all the participants received the 360-degree video-based condition in a counterbalanced way (1st narrative, 2nd 360-degree video-based or 360-degree video-based 2nd narrative), and we are only interested in the measures of embodiment, we do not distinguish between the conditions in testing the aims of the present study.
